# Comparing de novo transcriptome assembly tools in di- and autotetraploid non-model plant species

**DOI:** 10.1186/s12859-021-04078-8

**Published:** 2021-03-22

**Authors:** Silvia Madritsch, Agnes Burg, Eva M. Sehr

**Affiliations:** 1 AIT Austrian Institute of Technology, Center for Health and Bioresources, Tulln, Austria; 2 Center for Integrative Bioinformatics Vienna, Max Perutz Labs, University of Vienna, Medical University of Vienna, Vienna, Austria

**Keywords:** RNA-seq, De novo transcriptome assembly, Autotetraploid, Polyploid, Plants, TransLiG

## Abstract

**Background:**

Polyploidy is very common in plants and can be seen as one of the key drivers in the domestication of crops and the establishment of important agronomic traits. It can be the main source of genomic repatterning and introduces gene duplications, affecting gene expression and alternative splicing. Since fully sequenced genomes are not yet available for many plant species including crops, de novo transcriptome assembly is the basis to understand molecular and functional mechanisms. However, in complex polyploid plants, de novo transcriptome assembly is challenging, leading to increased rates of fused or redundant transcripts. Since assemblers were developed mainly for diploid organisms, they may not well suited for polyploids. Also, comparative evaluations of these tools on higher polyploid plants are extremely rare. Thus, our aim was to fill this gap and to provide a basic guideline for choosing the optimal de novo assembly strategy focusing on autotetraploids, as the scientific interest in this type of polyploidy is steadily increasing.

**Results:**

We present a comparison of two common (SOAPdenovo-Trans, Trinity) and one recently published transcriptome assembler (TransLiG) on diploid and autotetraploid species of the genera *Acer* and *Vaccinium* using *Arabidopsis thaliana* as a reference. The number of assembled transcripts was up to 11 and 14 times higher with an increased number of short transcripts for *Acer* and *Vaccinium*, respectively, compared to *A. thaliana.* In diploid samples, Trinity and TransLiG performed similarly good while in autotetraploids, TransLiG assembled most complete transcriptomes with an average of 1916 assembled BUSCOs vs. 1705 BUSCOs for Trinity. Of all three assemblers, SOAPdenovo-Trans performed worst (1133 complete BUSCOs).

**Conclusion:**

All three assembly tools produced complete assemblies when dealing with the model organism *A. thaliana*, independently of its ploidy level, but their performances differed extremely when it comes to non-model autotetraploids, where specifically TransLiG and Trinity produced a high number of redundant transcripts. The recently published assembler TransLiG has not been tested yet on any plant organism but showed highest completeness and full-length transcriptomes, especially in autotetraploids. Including such species during the development and testing of new assembly tools is highly appreciated and recommended as many important crops are polyploid.

**Supplementary Information:**

The online version contains supplementary material available at 10.1186/s12859-021-04078-8.

## Background

Polyploidy (often referred to as whole genome duplication, WGD) describes the presence of more than two sets of homologous chromosomes in a cell or an organism, is very common in higher plants and plays an important role in plant evolution, speciation and adaptation. It has been discovered that all flowering plants experienced at least two ancient polyploidization events [1] that led to new genes with novel functions [2]. In addition, recent polyploidization events in ferns, lycophytes, and many flowering plants resulted in the formation of neopolyploids, that partly established themselves as novel species [3]. Polyploidization is not only a key process happening in natural populations and species but plays a major role in crop breeding too. Important crops like potato, wheat, cotton, peanut or strawberry are polyploid organisms [4–7]. Two main categories of polyploidy are recognized: auto- and allopolyploidy. Whilst the first is the outcome of WGD within a species where a genome with multiple sets of homologous chromosomes is generated (e.g. AAAA in the case of an autotetraploid), allopolyploids originate through WGD that is based on the hybridization between species resulting in a genome with multiple sets of homoeologous chromosomes (each from a separate parental subgenome, e.g. AABB in allotetraploids) [8].

Besides the genomic repatterning that comes with WGD, it is known, that past WGD events and a subsequent high rate of maintaining pairs of duplicated genes throughout evolution led to a stable higher rate of duplicated genes in plant genomes, thereby changing the concentration of gene products resulting in gene dosage imbalances [9, 10]. Recent polyploidization events can have immediate phenotypic effects, such as increased cell size leading to an increase in biomass. Especially in allopolyploids, recent gene duplications can induce additional positive effects that are beneficial for plant breeding, such as heterosis and gene redundancy [11, 12]. The first effect causes more vigorous individuals while the latter protects polyploids from the deleterious effect of mutations [12]. But, many more mechanisms are known to be affected by WGD, as such it is well described, that the per-cell gene expression levels are increased in polyploids [13] and that stress related genes can change their expression pattern in polyploid species in comparison to their diploid counterparts [14, 15]. Additional hypotheses about transcriptional changes with regard to polyploidization are well reviewed in Doyle and Coate (2019) [16]. A further mechanism that is also influenced by polyploidization is alternative splicing (AS) [17]. In plants, more than 60% of intron-containing genes undergo AS [18, 19], whereby it is known that environmental stresses can cause even more splicing events [20]. As a modulator of gene expression, AS plays a crucial role in multiple biological processes during plant growth and development.

The analysis of gene expression through RNA sequencing (RNA-seq) is a well-established, commonly used method in both, basic and applied research to interpret functional elements of the genome and understand the formation of phenotypes, traits and the reaction to diseases and a changing climate [21]. The above described effects of polyploidization (high genomic complexity, gene duplications, dosage imbalances, affected AS) bring major challenges especially for the de novo transcriptome assembly that is applied commonly in non-model organisms when no reference genome is at hand. Besides the fact that the de novo assembly is already a complex task in diploids, due to the sequence similarity of transcripts that are isoforms, or are a product of allelic variants, close paralogs or homologs [22], this gets even more challenging in polyploids. While in allopolyploids an additional complexity level is given through the presence of homoeologous genes [23], autopolyploids usually have a high heterozygosity due to the nature of polysomic inheritance where e.g. four different alleles at a given locus with random pairing between each of the four chromosomes can result in nineteen genotypes. In contrast, allopolyploids usually show disomic inheritance that lead to bivalent chromosome formation resulting in a maximum of nine combinations for the given locus in the offspring [24, 25]. All these configurations (e.g. duplications, multiple alleles) cause extra branches and bubbles in the de Bruijn graph that is nowadays predominantly used to build the de novo transcriptome assemblies. Therefore, the graph structure can be ambiguous, and the represented isoforms can be challenging to resolve. As a result, a collapse of transcripts from genes belonging to one gene family (homologs), chimerism (the concatenation of two or more transcripts that may or may not be related) or redundancy (e.g. allelic sequences as separate loci) might occur more frequently [26, 27].

State of the art transcriptome assemblers were developed and tested in model organisms that lack high gene duplication rates or polyploidy levels [28–30] and thus, their evaluation in polyploids is scarce. Only a few studies focused on the comparison of transcriptome assembly strategies in polyploid species, among them only one including autotetraploids [31–33]. Despite those studies, there is a lack of cross-species analyses comparing the performances of these tools on multiple di- and polyploid species. To fill this gap and to provide a basic guideline for choosing the optimal de novo assembly strategy, we performed a comparison of two common (SOAPdenovo-Trans, Trinity) and one recently released transcriptome assembler (TransLiG) on diploid and autotetraploid non-model plant species, as the scientific interest in this type of polyploidy is increasing [34], but still, “studies about the regulation of genes on the four homologous chromosomes of autopolyploids have received little attention” [16].

As study organisms of choice, we focused on di- and autotetraploids from the plant genera *Acer* and *Vaccinium.* The genus *Acer* is an extremely diverse group containing over 120 species of various size, habit and ploidy level. Our *Acer* species of choice were sycamore maple (*Acer pseudoplatanus* L., 4×) and Norway maple (*Acer platanoides* L., 2×). Both species show a similar distribution pattern across Europe and are valuable hardwood species [35, 36]. Further, *Vaccinium* is a young and widespread genus with elevated rates of speciation in recent decades that led to the formation of about 450 species [37]. The genus includes blueberries, cranberries or lingonberries and consists of very complex polyploid species like *Vaccinium corymbosum* L., a highly economically relevant species in the food sector [37, 38]. In addition, to have a proven reference, a di- and an autotetraploid *Arabidopsis thaliana* L. genotype was included in our study. Among the tested tools, SOAPdenovo-Trans is a transcriptome assembler built on a genome assembler [29, 39], while Trinity [28, 39] was specifically developed for transcriptome assembly. The first was implemented and tested on transcriptome data of rice and mouse, the latter was established using transcriptome data of fission yeast. TransLiG is the most recently developed assembler, released in 2019, reviewed on human transcriptome data, with a special consideration to integrate the sequence depth and paired-end information to retrieve all the transcript-representing paths in splicing graphs [30]. To our knowledge, TransLiG has not been tested on any plant data so far.

## Methods

**Fig. 1:**
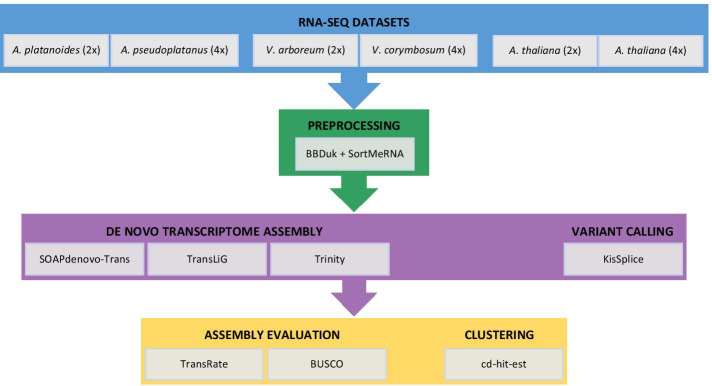
Pipeline for de novo transcriptome assembly evaluation

A schematic workflow of the data and tools used in this study is shown in Fig. 1.

### *Acer* sampling, RNA extraction, library preparation, and sequencing

For each of the two *Acer* species under investigation, *A. platanoides* L. (Norway maple, diploid = 2×) and *A. pseudoplatanus* L. (sycamore maple, tetraploid = 4×), three mature individuals were chosen for selection. The individuals are part of the living collection of woody plants of the Botanical Garden of the University of Vienna, Austria (Hortus Botanicus Vindobonensis, HBV), and can be identified through the following individual accession numbers: Norway maple tree IDs 37006, 30044, and IGF024; sycamore maple tree IDs PP001, 34011, and 32074 (cf. Additional file 1). Leaf material (comprising around five randomly selected small leaves per individual) was collected and immediately frozen in liquid nitrogen. Frozen leaf tissue was ground to a fine powder and from about 50–60 mg total RNA was extracted using TRIzol Reagent as described in Meng and Feldman (2010) [40]. Total RNA was sent on dry ice to the Next Generation Sequencing Facility at Vienna BioCenter Core Facilities (VBCF), Austria. There, the RNA was quality and quantity checked using Agilent’s Bioanalyzer. Library preparation was done using the NEB polyA enrichment kit, including stranded information and a cutout size between 300-800 bp, resulting in an individual median size of each library between 388 and 423 bp. All six mRNA libraries were sequenced on one lane on the HiSeq2500 PE150 in rapid mode. Sample information and sequence data are available at NCBI under the BioProject PRJNA662197.

### Additional data

Raw RNA sequence reads of three *V. arboreum* and three *V. corymbosum* individuals of the control group (pH 4.5) from the study by Payá-Milans et al. (2018) [32] were downloaded from https://www.ebi.ac.uk/ena, PRJNA353989. In that study, libraries were prepared using the Ribo-Zero™ rRNA Removal Kit on total RNA and ScriptSeq v2 RNA-Seq library preparation kit, and further sequencing was done in paired-end mode with a length of 101 bp and fr-strandness. The *A. thaliana* RNA-seq data generated by Zhang et al. (2019) [14] was downloaded from https://www.ebi.ac.uk/ena, PRJNA473317. In that case, total RNA was used for sequencing with standard Illumina protocols. A description of all the species used in this study is shown in Table 1, detailed meta data of each individual is provided in Additional file 1.

**Table 1:** Sample description

Species	Ploidy	Library type	Tissue	Selection	Strandness	Read length	Average M reads after preprocessing
*Acer platanoides*	Diploid	Paired-end	Leaves	poly-A	RF	150 bp	25.1
*Acer pseudoplatanus*	Tetraploid	Paired-end	Leaves	poly-A	RF	150 bp	29.1
*Vaccinium arboreum*	Diploid	Paired-end	Roots	rRNA depletion	FR	101 bp	12.9
*Vaccinium corymbosum*	Tetraploid	Paired-end	Roots	rRNA depletion	FR	101 bp	26.9
*Arabidopsis thaliana*	Diploid	Paired-end	Aerial parts	rRNA depletion*	non*	150 bp	21.6
*Arabidopsis thaliana*	Tetraploid	Paired-end	Aerial parts	rRNA depletion*	non*	150 bp	21.4

*Inferred from the data sets during our analyses

### De novo transcriptome assembly

Raw sequence reads were pre-processed for base quality (Q20 from left and right) and adapter content using BBDuk package from the software BBMap version 37.68 [41] as well as rRNA filtered using SortMeRNA version 3.0.3 (Kopylova 2012).

De novo transcriptome assemblies of all five species were performed with Trinity version 2.6.5 [28, 42], SOAPdenovo-Trans version 1.04 [29] as well as TransLiG version 1.3 [30] using all three biological replicates each (Table 1). Based on the library protocols that were used to sequence the RNA-seq data, Trinity assembly was performed with default values and –SS_lib_type RF for *Acer* data, FR for *Vaccinium* and no lib type for *Arabidopsis*. The replicates are indicated via the –samples_file parameter. Strandness for the TransLiG assemblies were indicated with the -m parameter. SOAPdenovo-Trans doesn’t offer a strand-specific option and thus analyses were run with default parameters. The maximum read length and the estimated average insert size was indicated in the SOAPdenovo-Trans config file. The insert size was estimated for each sample using raw sequenced reads and BBMerge [41] and averaged for each library type. For the input of SOAPdenovo-Trans and TransLiG the input files of replicates were concatenated. By default, the minimum contig length for Trinity and TransLiG is 201 bp while it is 100 bp for SOAPdenovo-Trans. For a more balanced evaluation of the assembly quality and as it was not possible to change the minimal contig length in SOAPdenovo-Trans, contigs smaller than 201 bp were removed from all SOAPdenovo-Trans assemblies.

### Genetic variants

The local transcriptome assembler KisSplice version 2.3.1 [43, 44] was used in default mode to call SNPs and short indels as well as to determine AS events on each species using pre-processed reads.

### Transcript clustering

To remove redundant and alternatively spliced transcripts, transcripts were clustered using cd-hit-est version 4.8.1 [45] with a sequence identity threshold of 95%. To focus specifically on the AS events, the number of unique genes were extracted for Trinity’s and TransLiG’s assemblies with the gene identifier that is saved in the transcript IDs. To investigate redundant transcripts in a stricter way, cd-hit-est was run with a sequence identity threshold of 95% (-c parameter), a length difference cutoff of 95% (-S), and an alignment coverage for the shorter sequence of 95% (-aS). To analyze the resulting clusters in detail the integrated perl-script plot_len1.pl was used.

### Transcriptome performance measures

Basic statistics were computed with TransRate version 1.0.3 [26] that uses SNAP sequence aligner [46]. Additionally, for the *Arabidopsis* assemblies, a comparison with the Ensembl *A. thaliana* reference cDNA set (release 47) and the reference protein set was performed using TransRate that includes CRB-BLAST [47]. The *Acer* assemblies were compared to the *Acer yangbiense* (assembly AYv1.1) protein set available at NCBI. The description of each output parameter of TransRate is given in detail on https://hibberdlab.com/transrate/metrics.html. Transcriptome completeness and contiguity was measured using BUSCO version 4.0.5 [48] in transcriptome mode with the eudicots.odb10 lineage dataset that includes 2,326 Benchmarking Universal Single-Copy Orthologs.

## Results

After pre-processing, the input size ranged from 11.3 million to 30.5 million reads per replicate. While the number of duplicated reads in *A. platanoides* samples was around 90% (estimated with FASTQC), duplications in other species varied between 35 and 78% (Additional file 1). The transcriptome wide GC content ranged from around 42% in *Acer* over 44% for *Vaccinium* to 47% for *Arabidopsis*. The amount of rRNA reads that were detected and filtered out was rather small (1%-4%) except for *A. platanoides* samples (7%-15%).

### Basic assembly evaluation

**Fig. 2:**
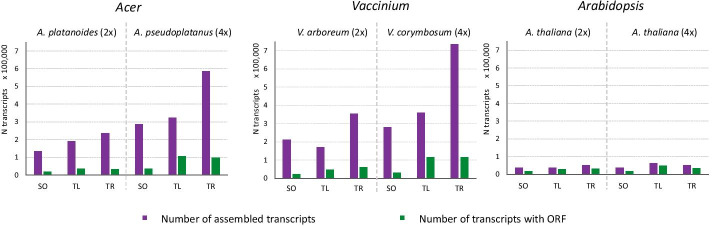
Number of assembled transcripts and ORFs. The number of assembled transcripts and the number of transcripts including an open reading frame (ORF) for each assembler (SO = SOAPdenovo-Trans, TL = TransLiG, TR = Trinity) is shown for the genera *Acer*, *Vaccinium* and *Arabidopsis* in diploid (2×) and autotetraploid (4×) species

**Fig. 3:**
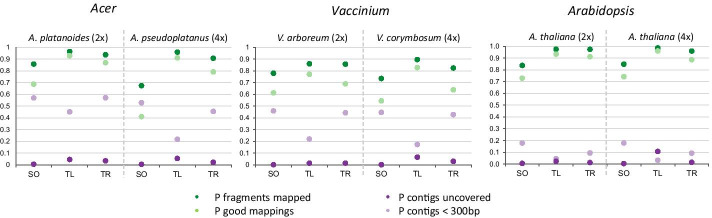
Basic assembly statistics. The proportion of reads (fragments) that mapped back to the assemblies (P fragments mapped), the proportion of good mappings i.e. both of the reads mapped on the same contig, with same orientation and without overlapping the ends of the contig (P good mappings), the proportion of transcripts with a length less than 300 bp (P contigs < 300 bp) and the proportion of contigs that are not covered by reads (P contigs uncovered) for each assembler (SO = SOAPdenovo-Trans, TL = TransLiG, TR = Trinity) is shown for the genera *Acer, Vaccinium* and *Arabidopsis* in diploid (2×) and autotetraploid (4×) species

Basic statistics of the assembly results (TransRate statistics) for each of the assemblers (SOAPdenovoTrans—SO, TransLiG—TL, Trinity—TR) and each species are visualized in Figs. 2 and 3 and described in detail in Additional file 2. The number of assembled contigs for the diploid *A. platanoides* ranged from 134,424 for SOAPdenovo-Trans, 190,917 for TransLiG to 235,011 for Trinity (Fig. 2). For the autotetraploid *A. pseudoplatanus* the number of contigs was 285,625 and 324,177 for SOAPdenovo-Trans and TransLiG, respectively, while it was almost the doubled amount for the Trinity assembly (587,214). Similar results were shown for the diploid *V. arboreum* (SO: 212,652—TL: 171,620—TR: 355,230) and the tetraploid *V. corymbosum* (SO: 280,852—TL: 361,369—TR: 735,465) (Fig. 2). The number of contigs for the *Arabidopsis* assemblies was just one-tenth compared to the other species. It ranged from 36,303 for SOAPdenovo-Trans to 51,431 for Trinity. In the autotetraploid *Arabidopsis* the number of contigs ranged from 36,442 for SOAPdenovo-Trans to 63,137 for TransLiG. On the other hand, the proportion of open reading frames (ORF) ranged from 0.11 to 0.33 for the *Acer* and *Vaccinium* assemblies while it was between 0.51 and 0.77 for all *Arabidopsis* assemblies (Fig. 2 and Additional file 2). Especially TransLiG showed the highest proportion of ORF in its assemblies.

The N50 sizes of the *Arabidopsis* assemblies were around 2000 bp while the N50 sizes of the other two genera (*Acer* and *Vaccinium*) were less, ranging from 513 to 995 bp, except for the TransLiG assemblies, were the N50 sizes were between 1539 bp (*V. arboreum*) and 2025 bp (*A. pseudoplatanus*) (Additional file 2). In general, smaller N50 sizes came with a high proportion of very short transcripts with a length of less than 300 bp. While the number of small transcripts in *A. thaliana* was between 3% (TransLiG, autotetraploid, 4×) and 10% (SOAPdenovo-Trans, di- and autotetraploid, 2× and 4×), the number of transcripts less than 300 bp was up to 46% in the *Vaccinium* assemblies and up to 57% in *A. platanoides* (Trinity and SOAPdenovo-Trans) (Fig. 3, Additional file 7). Additionally, the proportion of contigs that have >  = 50% estimated chance of being segmented (p_segmented) is higher in the *Acer* and *Vaccinium* assemblies (15–21%) compared to *Arabidopsis* (13–15%), with lower proportions in the autotetraploids (Fig. 3, Additional file 2).

According the number of reads (fragments) that mapped back to the assemblies and the number of good mappings (i.e. both of the reads mapped on the same contig, with same orientation and without overlapping the ends of the contig), the highest proportion was seen for Trinity and TransLiG assemblies in all species (Fig. 3 and Additional file 2). Especially for the autotetraploid species, TransLiG (AC: 0.96, VA: 0.90, AT: 0.99) outperformed Trinity (AC: 0.91, VA: 0.82, AT: 0.96) in the proportion of fragments that mapped and in the proportion of good mappings (TL: AC 0.91, VA 0.8, AT 0.96; TR: AC 0.79, VA 0.63, AT 0.89). The proportion of contigs uncovered was rather small for most of the assembly results. More than 5% of uncovered contigs (mean per-base read coverage of < 1) was only seen in the TransLiG assemblies of the autotetraploid species (Fig. 3).

### Assembly completeness

With regard to the completeness, we saw most complete assemblies (complete single plus complete duplicated BUSCOs) with TransLiG in both, diploid (AC: 1,613, VA: 1,368, AT: 2,115) and tetraploid (AC: 2,044, VA: 1,558, AT: 2,147) species (Additional file 3). Fewest complete BUSCOs were assembled for the SOAPdenovo-Trans assemblies in autotetraploid *Acer* (833) and autotetraploid *Vaccinium* (669). The completeness of *A. thaliana* assemblies was rather similar for all the assemblers, ranging from 1,932 to 2,147 complete BUSCOs. Focusing on the proportion of complete duplicated BUSCOs compared to the number of all complete BUSCOs, we saw the highest proportion in the TransLiG assemblies of tetraploid species (from 0.73 to 0.83, depending on species) and the least proportion in SOAPdenovo-Trans assemblies (from 0.08 to 0.29). On the other hand, most fragmented and missing BUSCOs were seen for SOAPdenovo-Trans assemblies in *Acer* and *Vaccinium* species.

### Comparison to the cDNA or protein reference

**Fig. 4:**
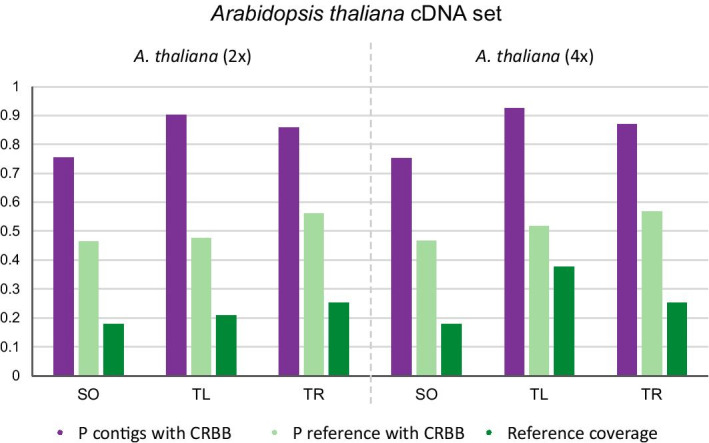
Comparison of *Arabidopsis thaliana* assemblies to the reference cDNA set. P contigs with CRBB—the proportion of contigs with a CRB-BLAST hit; P reference with CRBB—the proportion of references with a CRB-BLAST hit; Reference coverage—the proportion of reference bases covered by a CRB-BLAST hit

The comparison of *A. thaliana* assemblies to the reference cDNA showed that the proportion of transcripts that have a CRB-Blast hit with the reference ranged from 0.75 for SOAPdenovo-Trans up to 0.93 for TransLiG (Fig. 4). The proportion of the reference with a transcript hit was between 0.47 and 0.57 for all assemblies, with the highest values for the Trinity assemblies, 0.56 for diploid and 0.57 for tetraploid *A. thaliana*. The proportion of transcripts with a CRB-Blast hit and the proportion of reference cDNA with a transcript hit did not differ between di- and tetraploid *A. thaliana*. This was different for the per base reference coverage. In the diploid *A. thaliana* assemblies, the highest coverage of 0.25 was seen in the Trinity assembly, compared to 0.18 and 0.21 in SOAPdenovo-Trans and TransLiG, respectively. In the tetraploid *A. thaliana,* the far highest coverage was seen in the TransLiG assembly with 0.38 compared to 0.18 and 0.25 (Fig. 4 and Additional file 2).

The comparison of the *Arabidopsis* assemblies with the reference protein set showed that the proportion of contigs with a CRB-Blast hit for SOAPdenovo-Trans is significantly decreased to less than 0.4 for both di- and tetraploid *A. thaliana* (Additional files 4 and 2). The difference between the proportion of reference with a transcript hit and the per base reference coverage was small for all assemblers, in contrast to the comparison of *Acer* assemblies to the reference *A. yangbiense* protein set. For *Acer*, the proportion of contigs with a CRBB hit was between 0.09 and 0.28 with the highest values in the TransLiG assemblies (Additional files 4 and 2). A comparison for *Vaccinium* to a reference protein set was not conducive due to the lack of a reasonable protein set for any *Vaccinium* species.

### Genetic variants

AS events, SNPs and short indels were called with a local transcriptome assembler. The number of SNPs was similar in both di- and tetraploid *A. thaliana* samples with around 23,000 SNPs, the number for diploid *Acer* and *Vaccinium* was more than 100,000 SNPs, and the number for autotetraploid *Acer* and *Vaccinium* 571,648 and 351,211 SNPs, respectively (Additional file 5). The number of detected genetic variants in diploid *A. thaliana* is comparable with the number in autotetraploid *A. thaliana*. Regarding AS events and short indels (< 3nt), the least were found in diploid *V. arboreum* (8,706 and 6,700, respectively), and the most in autotetraploid *A. pseudoplatanus* (60,467 and 88,689, respectively) (Additional file 5).

### Transcript clustering

**Fig. 5:**
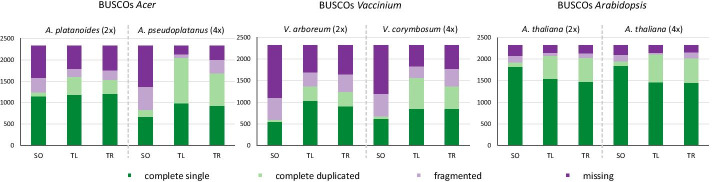
Assembled BUSCOs for clustered assemblies. The number of complete BUSCOs for each assembler clustered with cd-hit-est, 95% sequence identity threshold (SO = SOAPdenovo-Trans, TL = TransLiG, TR = Trinity), is shown for the assembled transcriptomes with genera *Acer*, *Vaccinium* and *Arabidopsis* in diploids (2×) and autotetraploids (4×)

To further investigate AS events and redundant transcripts, a clustering of the assembled transcripts was performed with cd-hit-est with a sequence identity threshold of 95%. The proportion of resulting representative transcripts was high in the SOAPdenovo-Trans assemblies (0.95–0.99) and very low in the TransLiG assemblies of autotetraploids (0.60–0.67) (Additional file 6). The further analysis of the completeness with BUSCO showed that in the *Vaccinium* assemblies the completeness even got little higher in most cases (up to + 6 complete BUSCOs) while the reduction in *Arabidopsis* assemblies was the highest (-4 to -36 complete BUSCOs) (Fig. 5 and Additional file 3). In general, the number of duplicated BUSCOs decreased in the clustered assemblies compared to the non-clustered ones. The proportion of duplicated BUSCOs in the *A. pseudoplatanus* TransLiG assembly decreased the most from 0.83 to 0.52 and in the autotetraploid *A. thaliana* from 0.75 to 0.31 (Fig. 5 and Additional file 3).

### Alternative splicing estimation

To estimate the amount of AS forms in the assemblies, both, Trinity’s and TransLiG’s integrated information of the gene ID within the transcript IDs showed that in general more isoforms per genes were present in the autotetraploids (1.5–1.8) compared to diploids (1.2–1.7) with the highest values in *A. thaliana*. In general, Trinity resulted in a stricter clustering than TransLiG (Additional file 6).

To investigate the number of transcripts that represent different alleles rather than true AS forms, cd-hit-est was run with stricter parameters, integrating transcript and alignment length information. Here, SOAPdenovo-Trans assemblies had the highest proportion of representative transcripts with 99% to 100% in di- and autotetraploid species, respectively, while the proportion in the TransLiG assemblies of the autotetraploids was between 83 and 85% (Additional file 7).

### Key findings

The key findings of this study were summarized for each assembler, averaged for all investigated species and provided in Table 2. TransLiG produced for both, di- and autotetraploid species, assemblies with the highest amount of reads that mapped back to the assembly in a sufficient way (0.88 and 0.90, respectively, SO: 0.68; 0.57, TR: 0.82; 0.77). Further, TransLiG had the lowest proportion of short transcripts (0.24 and 0.14), the highest amount of complete BUSCOs (1,699 and 1,916, SO: 1,266; 1,133 TR: 1,615; 1,705), and the lowest number of fragmented BUSCOs (188 and 132, SO: 328; 419, TR: 240; 276). Trinity assemblies on the other hand showed the highest protein reference coverage (0.45 and 0.51, respectively), but only slightly better than TransLiG (0.42 and 0.50). Comparing the *A. thaliana* assemblies to the complete cDNA reference set, highest reference coverage was seen for diploid *A. thaliana* assembled with Trinity (0.25 vs 0.18 and 0.21) while the reference coverage of tetraploid *A. thaliana* was the highest for the TransLiG assembly (0.38 vs 0.18 and 0.25). SOAPdenovo-Trans produced assemblies with the least proportion of uncovered bases (0.02) and the highest proportion of representative transcripts (0.97 and 1.00) using two different parameters for clustering. The lowest number of representative transcripts was seen for TransLiG in the autotetraploids (0.63 or 0.84 with stricter parameters). The number of assembled transcripts was similar for SOAPdenovo-Trans (0.13 and 0.20 million transcripts) and TransLiG (0.13 and 0.25) but significantly higher for Trinity (0.21 and 0.46). In general, the differences between the assembler diverged more within autotetraploid species compared to the diploid species.

**Table 2:** Summary of the key findings shown for each assembler

	Diploid species			Tetraploid species		
	SO	TL	TR	SO	TL	TR
Number of M transcripts	0.13 (0.09)	0.13 (0.08)	0.21 (0.15)	0.20 (0.14)	0.25 (0.16)	0.46 (0.36)
Proportion of transcripts < 300 bp	0.40 (0.20)	**0.24 (0.20)**	0.37 (0.25)	0.38 (0.18)	**0.14 (0.10)**	0.32 (0.20)
Proportion of good mappings	0.68 (0.05)	**0.88 (0.08)**	0.82 (0.10)	0.57 (0.16)	**0.90 (0.07)**	0.77 (0.12)
Proportion of bases uncovered	**0.02 (0.01)**	0.06 (0.01)	0.05 (0.01)	**0.02 (0.01)**	0.13 (0.01**)**	0.06 (0.02)
Complete BUSCOs	1,266 (667)	**1,699 (381)**	1,615 (410)	1,133 (714)	**1,916 (315)**	1,705 (340)
Fragmented BUSCOs	328 (177)	**188 (140)**	240 (156)	419 (238)	**132 (129)**	276 (145)
Reference coverage (proteins) excl. *Vaccinium*	0.35 (0.03)	0.42 (0.03)	**0.45 (0.01)**	0.34 (0.00)	0.50 (0.10)	**0.51 (0.09)**
Reference coverage of *A. thaliana* cDNA	0.18	0.21	**0.25**	0.18	**0.38**	0.25
Proportion of representative transcripts (cd-hit-est 95% sequence identity)	**0.97 (0.02)**	0.81 (0.04)	0.86 (0.02)	**0.97 (0.03)**	0.63 (0.04)	0.80 (0.10)
Proportion of representative transcripts (cd-hit-est strict*)	**1.00 (0.00)**	0.92 (0.00)	0.96 (0.01)	**1.00 (0.00)**	0.84 (0.01)	0.94 (0.01)

Mean (SD) values of all investigated diploid species (*A. platanoides*, *V. arboreum* and *A. thaliana* 2×) and all tetraploid species (*A. pseudoplatanus*, *V. corymbosum* and *A. thaliana* 4×) were calculated for SOAPdenovo-Trans (SO), TransLiG (TL) and Trinity (TR) assemblies. Bold values highlight the best performing assembler for each parameter and within each ploidy class

*Transcripts are clustered with cd-hit-est with 95% sequence identity (-c), 95% length difference cutoff (-S) and 95% alignment coverage (-aS)

## Discussion

Due to the lack of a comparative study of current de novo transcriptome assemblers including autotetraploid plant species, we analyzed representatives of three plant genera, *Acer*, *Vaccinium* and *Arabidopsis* using SOAPdenovo-Trans, Trinity, and the recently—in 2019—released assembler TransLiG.

### Assembler-independent transcript number variation

The generated de novo transcriptome assemblies showed differing numbers of transcripts for each genus independently from the assembler used. While all assemblers produced less than 65,000 transcripts for both, di- and autotetraploid *A. thaliana*, the number of transcripts in the *Acer* and *Vaccinium* assemblies was multiple times higher ranging from 134,424 up to around 355,230 in case of the diploids, and from around 280,852 to more than 735,465 in the autotetraploids. That tendency is surprising because the number of genes in some *Vaccinium* and *Acer* species is estimated only to be in the range or utmost twice the number of the 27,000 annotated genes in *A. thaliana* [37, 49–51]. Of course, one should be aware that this is only an estimation and could be underestimated for *Acer* and *Vaccinium* due to the lack of completely sequenced and annotated genomes for those genera [52]. Further, it is known that the number of genes in plant species can vary significantly even between closely related species [2, 9]. In addition, AS events come with an increase in the number of transcripts, a correlation, that can be seen in the polyploid samples analyzed in here but seemed not to have occurred more frequently in our diploid *Acer* and *Vaccinium* samples compared to diploid *A. thaliana* samples using different algorithms (KisSplice, Trinity and TransLiG). A large genetic distance between the replicates could theoretically also increase the number of assembled transcripts but should have been avoided especially using Trinity because replicates were indicated in the input file. We further observed a higher number of small contigs in *Acer* and *Vaccinium* most likely due to the presence of fragmented genes that are very low expressed in those species. Analyses of transcripts with similar length and a high sequence similarity showed way more redundant transcripts in *Acer* and *Vaccinium* than in *Arabidopsis.* These results and the higher number of detected SNPs with KisSplice suggest a higher heterozygosity in *Acer* and *Vaccinium* that may lead to additional contigs in Trinity or TransLiG using default parameters. However, a subset of the higher number of transcripts in *Acer* and *Vaccinium* might still be explainable due to an underestimation of proteins in those species [52].

### Non-model species show less complete transcriptomes

When we investigate the completeness and contiguity of the assembled transcriptomes there were more missing and fragmented transcriptomes assembled for *Acer* and *Vaccinium*. All assemblers performed very well in the model plant *Arabidopsis*, regardless of its ploidy level, but the results between the assemblers varied tremendously for *Vaccinium* and especially *Acer*, with one exception: only for the autotetraploid *A. pseudoplatanus* a similar amount of complete BUSCOs, as seen in *Arabidopsis,* could be assembled by TransLiG*.* After clustering of the assemblies (to reduce redundancy), the amount of complete (single + duplicated) BUSCOs did not change significantly compared to the unclustered assemblies. However, the distribution between the complete single and the complete duplicated changed, with an overall increase of complete single at the expense of complete duplicated BUSCOs. Thus, a species-specific analysis of the best appropriate similarity threshold to find the optimal balance between redundancy and completeness is recommended.

Differences in assembler performance within and among species have already been described in previous studies. Similar to our results, Hölzer et al. (2019) showed, that the completeness in *A. thaliana* or *E. coli* was similar for nine out of ten investigated assemblers (except BinPacker) ranging from 930 to 1,119 complete BUSCOs and 255 to 332, respectively, while it was quite varying in *H. sapiens* (1,682 to 4,106 complete BUSCOs) [39]. In accordance with the results from Payá-Milans et al. (2018) and Li et al. (2019), the completeness of the SOAPdenovo-Trans assembly was less than that of the Trinity assembly [32, 53]. In contrast to our study, where Trinity was in the midst in terms of the completeness, it showed the lowest completeness of the hexaploid sweet potato assembly compared to other investigated assembly tools [33]. As TransLiG is a recently developed assembler it was not integrated in any of those studies but outperformed all other assemblers within and among species in terms of completeness in our study.

Comparing the *A. thaliana* assemblies to the reference cDNA set, we saw that there was a discrepancy between the number of reference cDNA that had a hit with a transcript and the per base reference coverage, indicating that many transcripts could not be assembled in full length. Interestingly, TransLiG assembled many more full-length transcripts compared to the other assemblers but only in case of the autotetraploid *A. thaliana*. Focusing on the assembled proteins, the difference between the number of reference cDNA that had a hit with a transcript and the per base reference coverage was evanescent. Most proteins were assembled in full length for *A. thaliana* but in general we recognized missing ends in the untranslated regions. Noticeable was further the low amount of contigs that mapped to the reference *A. thaliana* protein set for SOAPdenovo-Trans. Due to the reason that this was not seen comparing the reference cDNA set, one could conclude that more local indel errors occurred that led to a change in the translated amino acids in the SOAPdenovo-Trans assemblies. For *Acer*, it seemed that even proteins were not assembled in full length. The highest proportion of transcripts that mapped to the cDNA reference was seen for the TransLiG assembly.

### TransLiG: a good option for de novo transcriptome assembly of autotetraploids

According to basic assembly statistics, the performance of each assembler was similar across the species. In general, for *Acer* and especially *Vaccinium* the proportion of fragments that mapped back to the assembly and the proportion of good mappings was little less than for *Arabidopsis*. According to those two parameters, TransLiG overall performed best while SOAPdenovo-Trans performed worst. In Payá-Milans et al. (2018), it is also seen that the proportion of reads mapped back to the assemblies was less for SOAPdenovo-Trans compared to Trans-ABySS and Trinity [32].

Focusing on the assemblers’ performance comparing diploid to autotetraploid organisms it needs to be pointed out that the polyploid *A. thaliana* genotype used in this study was synthetically generated through colchicine and has not undergone any evolutionary forces through time which might have shaped the autopolyploid *Acer* and *Vaccinium* species. Thus, it was not surprising that all assemblers performed similarly in the autotetraploid *A. thaliana* compared to the diploid *A. thaliana* samples. Also, the number of bubbles and extra branches in the de Bruijn graph did not increase significantly. Comparing the polyploid *Acer* and *Vaccinium* species to their diploid equivalents, all assemblers produced more contigs. Our results indicate, that this is likely the product of a higher rate of AS events as well as a higher proportion of sequence similarity (e.g. heterozygosity, paralogs). The former outcome is well supported through the known increase of AS events in polyploid plants during evolution [17]. With regard to sequence similarity, TransLiG showed the highest number of redundant transcripts while SOAPdenovo-Trans produced almost non-redundant transcripts. TransLiG, in particular, also showed a high number of redundant transcripts even for the synthetically generated tetraploid *A. thaliana*. In contrast to diploid organisms, where apart from homozygotes AA (reference alleles) and CC (alternative alleles) only one class of heterozygotes is expected (AC), in tetraploids, we might expect three different classes of heterozygotes AAAC (simplex), AACC (duplex) and ACCC (triplex) [54]. Besides this natural increase in heterozygosity a generally higher mutation rate [55] as well as an accelerated stress adaptation [7] might increase the redundancy effect in polyploids as well. Due to the reason that a high sequence similarity in polyploids might also be due to duplicated genes (paralogs) or a high heterozygosity among replicates [25] further studies using model organisms need to be performed to distinguish the effects of those phenomena in de novo assembly of polyploids in detail.

A higher number of fragmentations, in particular short fragments, seemed not to be the reason for the additional contigs in polyploid species. For all assembler the proportion of short contigs decreased in the autotetraploid species. Especially for the polyploids, TransLiG outperformed Trinity regarding good mappings, complete and fragmented BUSCOs. A reason could be that TransLiG better integrates the sequence depth and paired-end information into the assembly procedure and thus is able to assemble complex genomes with increased AS more accurately [30].

## Conclusions

In general, state of the art assemblers had much more difficulties in accurately assembling complex plant transcriptomes with high gene duplication rates (*Acer*, *Vaccinium*) than standard diploid model organisms (*A. thaliana*). The recently published assembler TransLiG had not been tested yet on any plant organism but showed highest completeness and full-length transcriptomes especially for the autotetraploid species in our study. Comparing the assemblies to the reference *Acer* and *Arabidopsis* protein sets, Trinity assemblies had the highest reference coverage, but only slightly better than TransLiG. SOAPdenovo-Trans assemblies performed worst for most of the investigated metrics in di- and autotetraploids but had the lowest number of uncovered bases and the least redundancy. On the other hand, Trinity and TransLiG produced a high number of redundant transcripts for complex and autotetraploid species where transcript clustering after assembly is highly recommended.

We further saw that all investigated assembly tools produced complete assemblies when dealing with the model organism *A. thaliana* independently of its ploidy level, but their performances differed extremely when it came to assemble complex and polyploid non-model plant species. Including such species during the development and testing of new assembly tools is highly appreciated and recommended as many economically important crops show high sequence similarity and various levels of polyploidy.

## Supplementary Information

**Additional file 1:** Detailed metadata description of individual samples.

**Additional file 2:** Detailed TransRate results including the comparison to the respective reference data.

**Additional file 3:** Detailed BUSCO results for each assembler and species.

**Additional file 4:** Comparison of *Arabidopsis thaliana* and *Acer* assemblies to the respective reference protein sets.

**Additional file 5:** Number of genetic variants for each species computed with KisSplice.

**Additional file 6:** Analysis of transcripts after different clustering methods

**Additional file 7:** Analysis of clusters produced with cd-hit-est (95% sequence identity (-c), 95% length difference cutoff (-S), 95% alignment coverage (-aS)

## Data Availability

Raw sequence data of *Acer* samples is available at NCBI Sequence Read Archive (SRA, http://www.ncbi.nlm.nih.gov/sra/) under the BioProject PRJNA662197. Other datasets analysed in the current study concerning *Vaccinium* and *Arabidopsis* are available under the BioProject PRJNA353989 and PRJNA473317.
