# Supplementary material for: Comparing de novo transcriptome assembly tools in di- and autotetraploid non-model plant species
Source: BMC Bioinformatics. 2021 Mar 22;22:146. doi: 10.1186/s12859-021-04078-8 (PMC7986043; doi:10.1186/s12859-021-04078-8)
Supplement: Supplementary file 4 — Additional file 4: Comparison of Arabidopsis thaliana and Acer assemblies to the respective reference protein sets. [file 12859_2021_4078_MOESM4_ESM.pptx]

## Slide 1
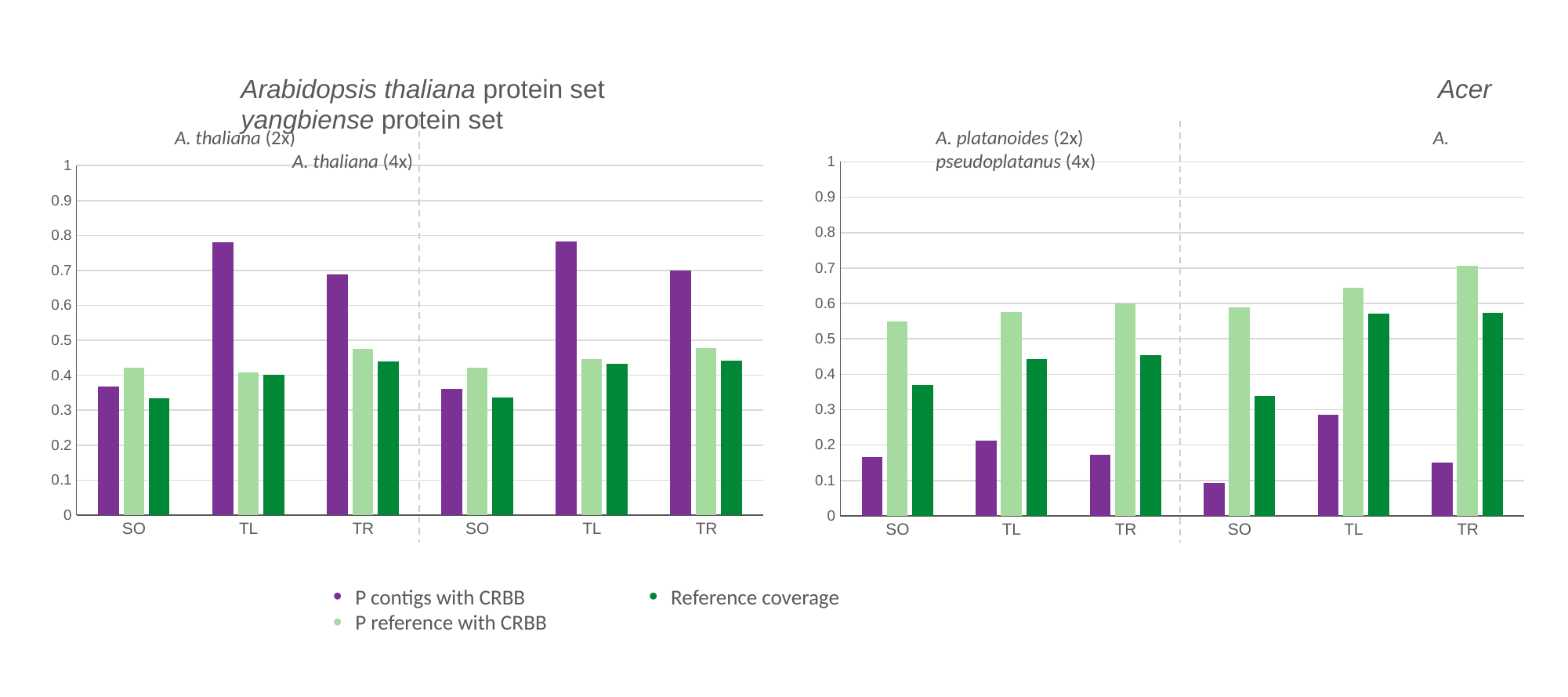

Arabidopsis thaliana protein set							 Acer yangbiense protein set
### Chart
| Category | P contigs with CRBB | P reference with CRBB | Reference coverage |
|---|---|---|---|
| SO | 0.16507 | 0.54785 | 0.36894 |
| TL | 0.21038 | 0.57447 | 0.44229 |
| TR | 0.17216 | 0.59912 | 0.45291 |
| SO | 0.09238 | 0.58828 | 0.33802 |
| TL | 0.28494 | 0.64364 | 0.57081 |
| TR | 0.14875 | 0.7059 | 0.57333 |A. thaliana (2x)	 			A. thaliana (4x)
A. platanoides (2x)	 		 A. pseudoplatanus (4x)
### Chart
| Category | P contigs with CRBB | P reference with CRBB | Reference coverage |
|---|---|---|---|
| SO | 0.3674 | 0.42147 | 0.33365 |
| TL | 0.77894 | 0.40748 | 0.40001 |
| TR | 0.68789 | 0.47338 | 0.43862 |
| SO | 0.35978 | 0.41938 | 0.33536 |
| TL | 0.7814 | 0.44562 | 0.43174 |
| TR | 0.69843 | 0.47656 | 0.44166 |P contigs with CRBB
P reference with CRBB
Reference coverage
